# Effects of highland environments on clonal diversity in aquatic plants: An interspecific comparison study on the Qinghai-Tibetan Plateau

**DOI:** 10.3389/fpls.2022.1040282

**Published:** 2022-10-20

**Authors:** Zhigang Wu, Zhong Wang, Dong Xie, Huijun Wang, Aiwen Zhao, Yalin Wang, Hanling Wang, Xinwei Xu, Tao Li, Jindong Zhao

**Affiliations:** ^1^ The State Key Laboratory of Freshwater Ecology, Institute of Hydrobiology, Chinese Academy of Sciences, Wuhan, China; ^2^ Department of Ecology, College of Life Sciences, Wuhan University, Wuhan, China; ^3^ School of Science, Tibet University, Lhasa, China; ^4^ Co-Innovation Center for Sustainable Forestry in Southern China, College of Biology and the Environment, Nanjing Forestry University, Nanjing, China; ^5^ The National Wetland Ecosystem Field Station of Taihu Lake, National Forestry Administration, Suzhou, China; ^6^ University of Chinese Academy of Sciences, Beijing, China; ^7^ Xi’an Jiaotong-Liverpool University, Suzhou, China; ^8^ State Key Laboratory of Protein and Plant Genetic Engineering, College of Life Sciences, Peking University, Beijing, China

**Keywords:** clonality, elevation, freshwater macrophyte, genetic diversity, linear model, wetland

## Abstract

Clonal reproduction is one of the most distinctive characteristics of plants and is common and diverse in aquatic macrophytes. The balance between sexual and asexual reproduction is affected by various conditions, especially adverse environments. However, we know little about clonal diversity of aquatic plants under suboptimal conditions, such as at high altitudes, and having this information would help us understand how environmental gradients influence patterns of clonal and genetic variation in freshwater species. The microsatellite data of four aquatic taxa in our previous studies were revisited to estimate clonal and genetic diversity on the Qinghai-Tibetan Plateau. Clonal diversity among different genetic groups was compared. Local environmental features were surveyed. Beta regressions were used to identify the environmental factors that significantly explained clonal diversity for relative taxon. The level of clonal diversity from high to low was *Stuckenia filiformis* > *Hippuris vulgaris* > *Myriophyllum* species > *Ranunculus* section *Batrachium* species. A positive correlation between clonal and genetic diversity was identified for all taxa, except *H. vulgaris*. Clonal diversity was affected by climate in *S. filiformis* and by the local environment in *H. vulgaris*. For *Myriophyllum* spp., low elevation and high sediment nutrition were significant for sexual recruitment. The environmental effects on clonal diversity were not significant in *R.* sect. *Batrachium* spp. Clonal diversity of aquatic plants is moderate to high and varies greatly in highlands. The effects of breeding systems and environmental factors on the patterns of clonal variation were identified. Elevational gradients, climates and local conditions play different roles in clonal diversity among relative taxon. Our results highlight the importance of sexual recruitment in alpine aquatic plant populations and the influence of environmental factors on the genetic patterns in freshwater species at local and regional scales.

## Introduction

Clonal reproduction is one of the most distinctive features of plants, especially wetland species (Sosnová et al., 2010). Plants can rapidly expand to occupy local niches, and exploit resource limited habitats *via* clonal integration (Ouborg et al., 1999; Liu et al., 2016). In extremely adverse habitats, vegetative growth and propagation are cost effective life-history strategies for population maintenance, responding to a risk of sexual reproduction failure (Weppler et al., 2006; Vallejo-Marín et al., 2010; Reisch et al., 2020). The balance of sexual and clonal reproduction is vital to clonal diversity within populations, which is affected by ecological heterogeneity in natural plant populations (Thompson and Eckert, 2004; Frei et al., 2014). Generally, suboptimal conditions, such as limited availability of light, water and nutrition, might promote plants to switch from sexual to clonal reproduction (Xie et al., 2016).

Compared to that in terrestrial species, clonal reproduction is essential for population maintenance in hydrophytes, when ecological conditions for sexual reproduction in aquatic environments are severe (Barrett et al., 1993; Grace, 1993; Santamaría, 2002). Aquatic macrophytes exhibit impressive variation in their breeding systems and asexual reproduction types, which are superior on efficient dispersal by water flow, and enhanced long-lived survival (Sculthorpe, 1967; Barrett et al., 1993; Yakimowski and Barrett, 2014; Eckert et al., 2016). Although clonal reproduction is prevalent and dominant in many aquatic taxa (Barrett et al., 1993; Santamaría, 2002), the evolutionary advantages of sexual reproduction for genetic diversity, adaptative potential, and long-distance dispersal capacity have rarely been reduced (Eckert, 2002; Silvertown, 2008; Vallejo-Marín et al., 2010; Barrett, 2015). Studies on the evolutionary and ecological consequences and interplay of reproduction systems in aquatic plants remain challenging, although the topics have been well to depth in some species (e.g., *Sagittaria latifolia*, Van Drunen and Dorken, 2012 and *Butomus umbellatus*, Thompson and Eckert, 2004). To date, we know little about how the environment affects the balance between or allocation to sexual and asexual reproduction (Li, 2014).

Alpine ecosystems are characterized by stressful environments, which are selective to the breeding traits of plants (Körner, 2003; Sun et al., 2014). It has been suggested that more clonal plants can be found with increasing altitude, and a combination of sexual and clonal reproduction is common in the proliferation of alpine plants (Peck et al., 1998). The steep gradient of environmental factors in highland areas, such as temperature, precipitation, and sediment fertility (Körner, 2003), also make highlands an ideal place to study the diversity patterns of clonal plants related to these environmental factors (Halassy et al., 2005). The evolutionary histories of aquatic plants in alpine regions have been revealed in some species (Lu et al., 2016; Du and Wang, 2016), whereas few of them have focused on the patterns of clonal variation (Liu et al., 2014; Sun et al., 2014). Previous studies have implied that the sexual reproduction of aquatic plants in high elevation regions is not rare (Wang, 2009; Wu et al., 2016; Wu et al., 2022), but there is a lack of comparative studies on multiple species combined with environmental factors.

The amount of sexually and asexually produced offspring affects genetic variation, gene dispersal and biogeography in aquatic plants (Barrett et al., 1993; Eckert et al., 2016). Therefore, understanding how environments influence reproduction patterns is crucial for understanding the regional biodiversity pattern of wetland ecosystems. Through interspecific comparisons, we may obtain evidence about sexual reproduction limited by certain ecological factors (Honnay and Jacquemyn, 2008). In the present study, we focused on four common taxa of aquatic plants on the Qinghai-Tibetan Plateau (QTP), which is the highest plateau in the world. All focus species reproduce through both sexual and asexual means and present various distribution patterns across broad elevational range (Wang, 2003). Because of twisting branches and invisible underground connections among vegetative structure, it is difficult to distinguish the genets and ramets of aquatic plants through field work. The molecular genotyping data obtained in our previous studies were revisited to evaluate the clonal diversity within populations of relative taxon (Lu et al., 2016; Wu et al., 2019; Wu et al., 2020; Wu et al., 2022). We also measured the local environmental conditions of aquatic habitats and considered climatic factors as ecological variables to model their influences on clonal variation. We aimed to reveal the influence of environmental factors on the sexual reproduction and genetic diversity of aquatic plants in highland areas.

## Materials and methods

### Species and population genetic data

#### Myriophyllum

*Myriophyllum* L., also called watermilfoil, belongs to Haloragaceae, which is one of most polymorphic taxa of plants and varies from land shrubs to freshwater macrophytes. There are 63 apparent species in this genus, which are mainly distributed in Australia (Moody and Les, 2010). On the QTP, three *Myriophyllum* species have been recorded. The maximum elevation of *M. verticillatum* is approximately 3000 m, and therefore, we did not choose this plant for the present study. *M. spicatum* and *M. sibiricum* are submerged macrophytes and closed related with a widespread distribution, commonly occurring on the QTP. These species present a mixed reproduction system propagating through both sexual and vegetative propagules (mainly by fragments) (Aiken et al., 1979). Microsatellite markers have been used for hybrid identification in our previous work, and genetic data are in publicly available on Dryad (doi: 10.5061/dryad.c418b). Only pure populations were involved in the present work (11 microsatellite loci, 307 individuals from 21 populations).

#### Hippuris

*Hippuris* L. is a monogeneric genus of the aquatic clade in Plantaginaceae. *H. vulgaris* has a circumboreal distribution across the Northern Hemisphere and also occurs on the QTP, as its southern range. The plant is perennial mainly *via* rhizome and monoecious, bisexual and protogynous flowers. The germination rate of its seeds is important for sexual recruitment (Good, 1924). The plant is amphibious, presenting an emerged form in shallow water or near the shore and submersion in deep lentic water or rivers. The genetic and geographical structure of *H. vulgaris* on the QTP has been well studied (Chen et al., 2013; Lu et al., 2016), and microsatellite data in our previous study are available at LabArchives (Lu et al., 2016), doi: 10.6070/H4K64G3F) and were revisited in the present study (9 microsatellite loci, 227 individuals from 23 populations).

#### Ranunculus section Batrachium

*Ranunculus* section *Batrachium* (Ranunculaceae) is a diverse aquatic or semiaquatic group comprising over 30 species (Cook, 1966; Wiegleb et al., 2017). Plants are ecologically varied from amphiphytes to hydrophytes with laminar or capillary leaves (Wiegleb et al., 2017). Self-compatibility has commonly been reported in these taxa (Hong, 1991; Wang, 2009). *Ranunculus* sect. *Batrachium* comprises three distinct species of hydrophytes on the QTP, while two of them, *R. subrigidus* and *R. trichophyllus*, are frequently found across the plateau (Wang, 2009; Chen et al., 2014; Wu et al., 2019; Wu et al., 2022). The two species are both widely distributed across Eurasia and North America, and hybridization between them occurs (Wiegleb et al., 2017; Wu et al., 2022). Genetic analysis was performed on their QTP populations by microsatellite markers, and the data were obtained on DRYAD (doi: 10.5061/dryad.gxd2547md). Hybrid populations were precluded when the effects of genetic admixture were not considered in this study, microsatellite data of 16 loci on 348 individuals from 23 populations were used.

#### Stuckenia

*Stuckenia* is a genus of the aquatic group Potamogetonaceae. The diversity centre of the genus is in the highlands of Central Asia and the adjacent northern regions, including the QTP (Kaplan, 2008). *Stuckenia filiformis* is one of most widespread submerged plants on the QTP (Wang, 2003) and is also common in cold water regions of America and Eurasia (Kaplan, 2008). The species mainly reproduces *via* seeds or a variety of vegetative means (turions, subterranean rhizomes and tubers). The spatial genetic pattern of *S. filiformis* was quantified on the QTP previously (Du and Wang, 2016; Wu et al., 2020), and raw data of microsatellite alleles are currently being revisited (7 microsatellite loci, 338 individuals from 21 populations, **Supplementary Data 1**).

### Clone identification and clonal diversity

The index of clonal diversity estimated *via* molecular markers indicates the proportion of individuals derived from sexual and asexual reproduction. The index could be more adequate for reflecting the contributions of sexual and asexual reproduction to population regeneration, especially in the case of few allothogenic genotypes or under obvious selection pressure (Arnaud-Haond et al., 2007). The number of genets was not calculated in all previous studies, while clonal diversity was estimated in none. It might overestimate the clonal diversity when a threshold for minimum genetic distance between genets was ignored. Here, clone identification was reperformed. Reliability of subsampling of ramets and number of loci was confirmed using the R package RClone (Bailleul et al., 2016, **Supplementary Figure 1**). The threshold distance distinguishing pairs of ramets belonging to the same genets was determined based on frequency distribution pattern of pairwise genetic distances in Genodive version 3.0 (infinite allele model, Arnaud-Haond et al., 2007; Meirmans, 2020), which is also suitable for polyploids (*Myriophyllum* spp.). Pairwise Bruvo distance, which is irrespective of ploidy level, was also used for genet discrimination, calculated in R package poppr (Bruvo et al., 2004; Kamvar et al., 2014) and POLYSAT (for polyploidy data, Clark and Jasieniuk, 2011).

The clonal and genetic diversity were determined using Genodive. The number of ramets (nr), number of genotypes (ng), richness index [R= (ng-1)/(nr-1)] and Simpson complement unbiased D (Peet, 1974) were used for the evaluation of clonal diversity in the four taxa. Jackknife analysis were used to test the sample sizes are appropriate for index estimation. The variance in clonal index D decreased with increasing subsample size and leveled off below the number of sampled ramets in most populations. Gene diversity (heterozygosity within populations, Hs, Nei, 1987) was estimated, and the index assumed Hardy-Weinberg equilibrium and corrected for sampling bias.

**Figure 1:**
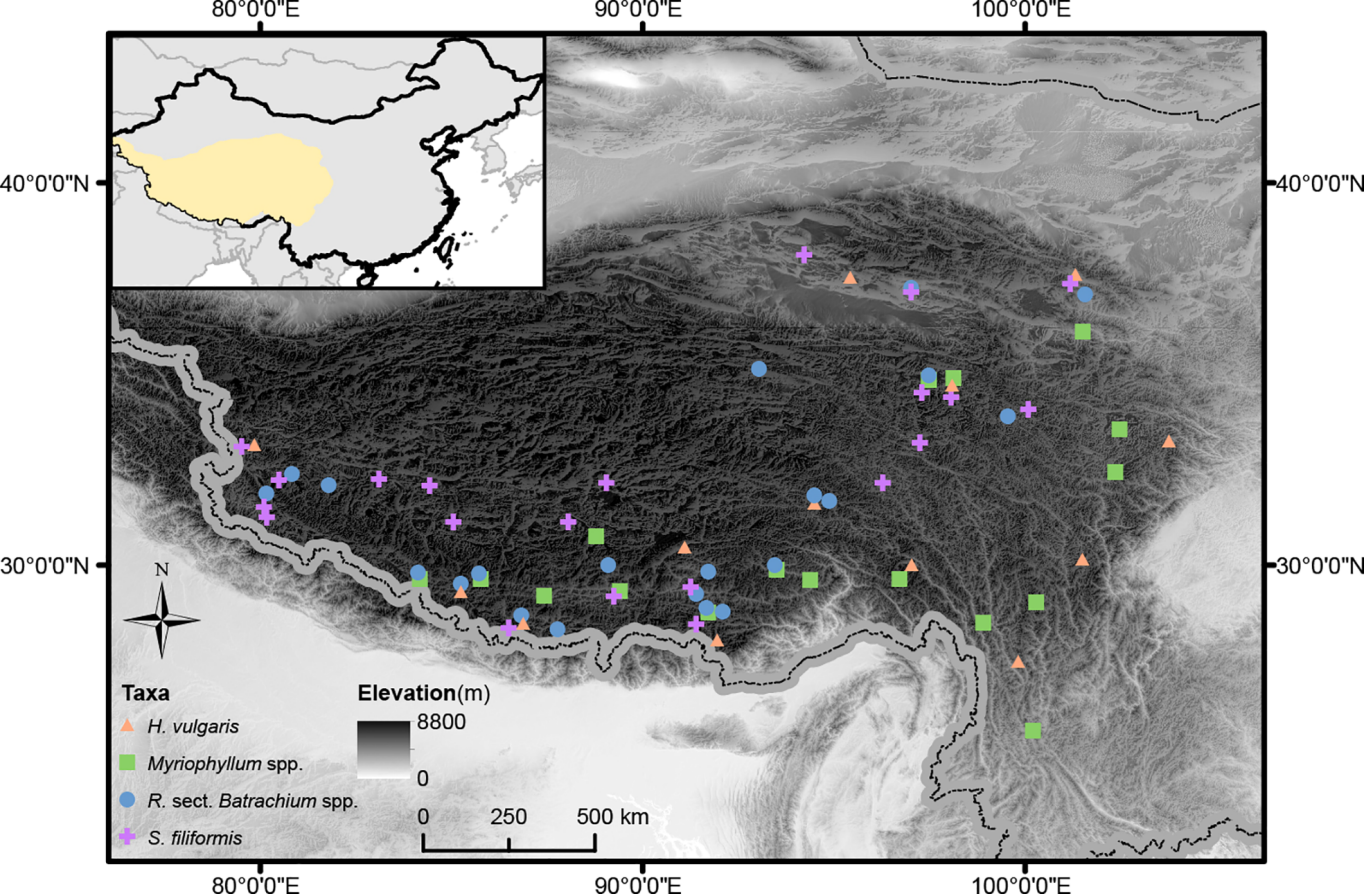
Geographic distribution of study sites for target taxa on the Qinghai-Tibetan Plateau. The elevation of the study region is visually displayed.

Populations in lotic sites, which likely to be monomorphic, or with small sample sizes were removed (Pollux et al., 2007; Kaplan, 2008). The clonal assignments generated from different programs were slightly different (**Supplementary Table 1**), we used the results of 75 populations from the four taxa evaluated in Genodive for subsequent analysis (**Figure 1**). The clonal diversity was mainly determined by the proportion of sexual and asexual offspring, which did not fit a normal distribution. We therefore applied nonparametric statistical tests (Wilcox and Kruskal−Wallis tests) to compare the clonal diversity among taxa and among genetic clusters within each taxon. For *Myriophyllum* spp. and *R.* sect. *Batrachium* spp., the two species with intercrossing, were treated as two genetic clusters. Genetic clades within *H. vulgaris* and *S. filiformis* suggested in Lu et al. (2016) and Wu et al. (2020) were recruited for the tests.

### Environmental data and linear regression

Climatic data of the studied sites were obtained from WorldClim with a resolution of 1 km (Fick and Hijmans, 2017, Bio1, annual temperature, AT and Bio4, temperature seasonality, TS). The local habitat features of the survey sites were quantified by the properties of the water and sediment during previous field work on *R.* sect. *Batrachium* spp. and *S. filiformis*. For *H. vulgaris* and *Myriophyllum* spp., sample sites were resurveyed for water and sediment features during 2015-2016. The pH and salinity (Sal) of the undisturbed aquatic habitat were measured using a handheld multiparameter metre (PROPLUS, YSI, USA). The two waterbody parameters were stable for years if the hydrology did not change obviously (Stephens, 1990; Koinig et al., 1998). Three 20 cm-deep sediment samples were collected before removing the covered plant tissue at each site. The total nitrogen concentration (TN) and carbon concentration (TC) in the air-dried sediment were determined with a CN elemental analyser (Vario MACRO cube, Elementar, Germany). The total phosphorus of the sediment (TP) was measured according to the molybdate/stannous chloride method after the samples were digested with H_2_SO_4_–H_2_O_2_–HF (Sparks, 1996).

Beta regressions with a logistic link function were used to identify the environmental factors that explained the clonal diversity of aquatic plants across the QTP regions. The beta regression model, an extension of generalized linear models, was suitable for situations in which dependent variables (clonal diversity index R and D) were asymptotic in the range between 0 and 1 (Cribari-Neto and Zeileis, 2010).

The best models with significantly important explanatory variables influencing clonal diversity were chosen based on the second-order Akaike information criterion (AIC) among all model combinations. A bias reduction for a small sample size was performed (Grün et al., 2012). We ranked the suggested models by AIC differences (ΔAIC) to the best model (AIC min). Akaike weights derived from AIC differences and pseudo *R* ^2^ values were also estimated for each model (Ferrari and Cribari-Neto, 2004). The candidate models were kept by the threshold value of ΔAIC < 2.0 to the best one, which was considered to have similar statistical confidence (Burnham and Anderson, 2004). We also precluded the models if the effect of any explainable factors was not significant. The relative importance of each environmental variable was evaluated by the sum of model Akaike weights over all models. The values of relative importance for particular variables ranged from 0 (not selected in any of the models) to 1 (selected in all models). The observation of 0 and 1 in clonal diversity would lead to failed fitting algorithms in beta regression; therefore, we applied a data transformation before modelling according to (Smithson and Verkuilen, 2006).

Pearson’s two-tailed correlation was firstly conducted to remove correlated environmental variables, which may bias regression analyses. If two variables were highly correlated (*r* > 0.7, *p* < 0.01), then we kept the one that resulted in a better fit model. We fit the models for each taxon because clonal diversity was significantly different in the four taxa but was not influenced by genetic divergence within taxa (see Results). All statistical analyses were conducted in R 4.1.2 (R Core Team, 2021). Beta regression was modelled in the R package ‘betareg’ (Cribari-Neto and Zeileis, 2010), and model selections were performed with the R package ‘MuMIn’ (Bartoń, 2022).

## Results

### Clonal and genetic diversity

The frequency distribution of genetic distances among multilocus genotypes was observed to follow a unimodal distribution when estimated with different distance methods, and relative distance thresholds of clone assignment were set for the four taxa (**Supplementary Figures 2**, **3**). The number of genotypes, clonal richness and Simpson diversity ranged from 1 to 11, 0 to 0.909, 0 to 0.985 for *H. vulgaris*; 1 to 14, 0 to 0.909, 0 to 0.985 for *Myriophyllum* spp.; 1 to 14 (1 to 12 discriminated based on Bruvo distance), 0 to 0.75, 0 to 0.944 for *R.* sect. *Batrachium* spp.; and 4 to 18 (5 to 19 discriminated based on Bruvo distance), 0.294 to 1, 0.562 to 1 for *S. filiformis*, respectively (**Supplementary Table 1**). The two clonal diversity indices were highly correlated (**Supplementary Figure 4**). The situation in which all sampled individuals came from an identical genet was found in 2, 4 (5 discriminated based on Bruvo distance), 8 and 0 populations of *H. vulgaris*, *Myriophyllum* spp., *R.* sect. *Batrachium* spp. and *S. filiformis*, respectively. The mean clonal diversity was highest in *S. filiformis* (R=0.668, D=0.875), while the lowest clonal diversity was in *R.* sect. *Batrachium* spp. (R=0.167, D=0.287) (**Table 1**).

**Table 1:** Information on the number and elevation range of sampled populations, average number of ramets and genets and clonal diversity for relative taxon.

Taxon	No. of Pop	Elev	mean nr	mean ng	mean R/D	median R/D
*Hippuris vulgaris*	16	2689-4810	11.56	4.688	0.360/0.587	0.348/0.711
*Myriophyllum* spp.	18	1954-5111	15.28	4.222	0.253/0.384	0.147/0.350
*Ranunculus* section *Batrachium* spp.	21	2628-5111	15.90	3.429	0.167/0.287	0.100/0.195
*Stuckenia filiformis*	21	2703-4913	16.10	11.333	0.668/0.875	0.733/0.917

Pop, population; Elev, elevation range (m); nr, number of ramets; ng, number of genets; R, index of clonal richness; D, Simpson complement unbiased clonal diversity.

**Figure 2:**
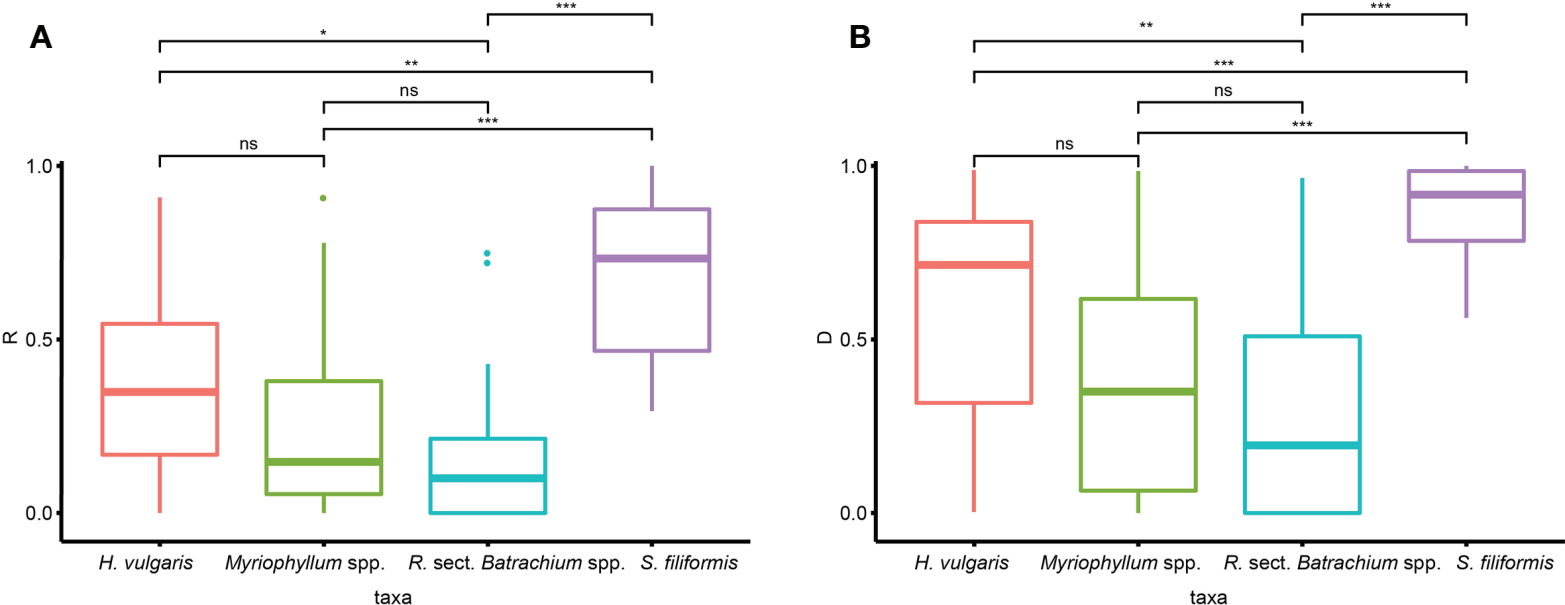
Variation in clonal diversity [**(A)** clonal index R, **(B)** clonal index D] among different taxa (ns, *p* ≥ 0.05; *, 0.05 < *p* ≤ 0.01; **, 0.01 < *p* ≤ 0.001; ***, *p* < 0.001).

The clonal diversity was significantly different among taxa, as suggested by Kruskal−Wallis tests (R: *p*<0.001, D: *p*<0.001). Wilcon tests performed for each species pair suggested that the clonal diversity of *S. filiformis* was significantly higher than that of the other three taxa, while *R.* sect. *Batrachium* spp. had significantly lower clonal diversity than *H. vulgaris* (**Figure 2**). There was no significant difference in clonal diversity among genetic clusters in either taxon (**Supplementary Table 2**).

The within-population genetic diversity Hs ranged from 0.056 to 0.365, 0.008 to 0.241, 0.079 to 0.338 and 0.232 to 0.563 for *H. vulgaris*, *Myriophyllum* spp., *R.* sect. *Batrachium* spp. and *S. filiformis*, respectively. Considering that the number and polymorphism of markers were different among the four taxa, differences in within-population gene diversity were not compared. A positive relationship between clonal variation and genetic diversity was suggested as occurring in *Myriophyllum* spp., *R.* sect. *Batrachium* spp. and *S. filiformis* but not in *H. vulgaris* (**Supplementary Figure 5**).

### Influence of environments on clonal diversity

Most sampling sites were alkaline and freshwater, and their pH values varied from 7.4 to 10.6 (**Supplementary Table 3**), and the pH of the sampled sites significantly increased with increasing altitude (**Supplementary Figure 6**). No more than 10 samples had salinities exceeding 0.5% (**Supplementary Table 3**), and *Myriophyllum* spp. and *S. filiformis* showed good tolerance to brackish water on the QTP. The TN and TP contents of the sediment varied from 0.49 to 8.88 and 0.10 to 0.87 mg/g, respectively, and the TC concentration was 43.08 mg/g on average and positively correlated with TN (**Supplementary Table 3** and **Supplementary Figure 6**). No significant relationship was found between sediment quality and altitude at the sample sites of the present work (**Supplementary Figure 6**). We included the pH and Sal of water, TC, TN and TP contents of sediment, climatic variables AT and TS, and elevation as the explanatory factors of the variation in clonal diversity along the environmental gradient, as suggested by the Pearson’s correlation analysis (**Supplementary Figure 6**).

**Figure 3:**
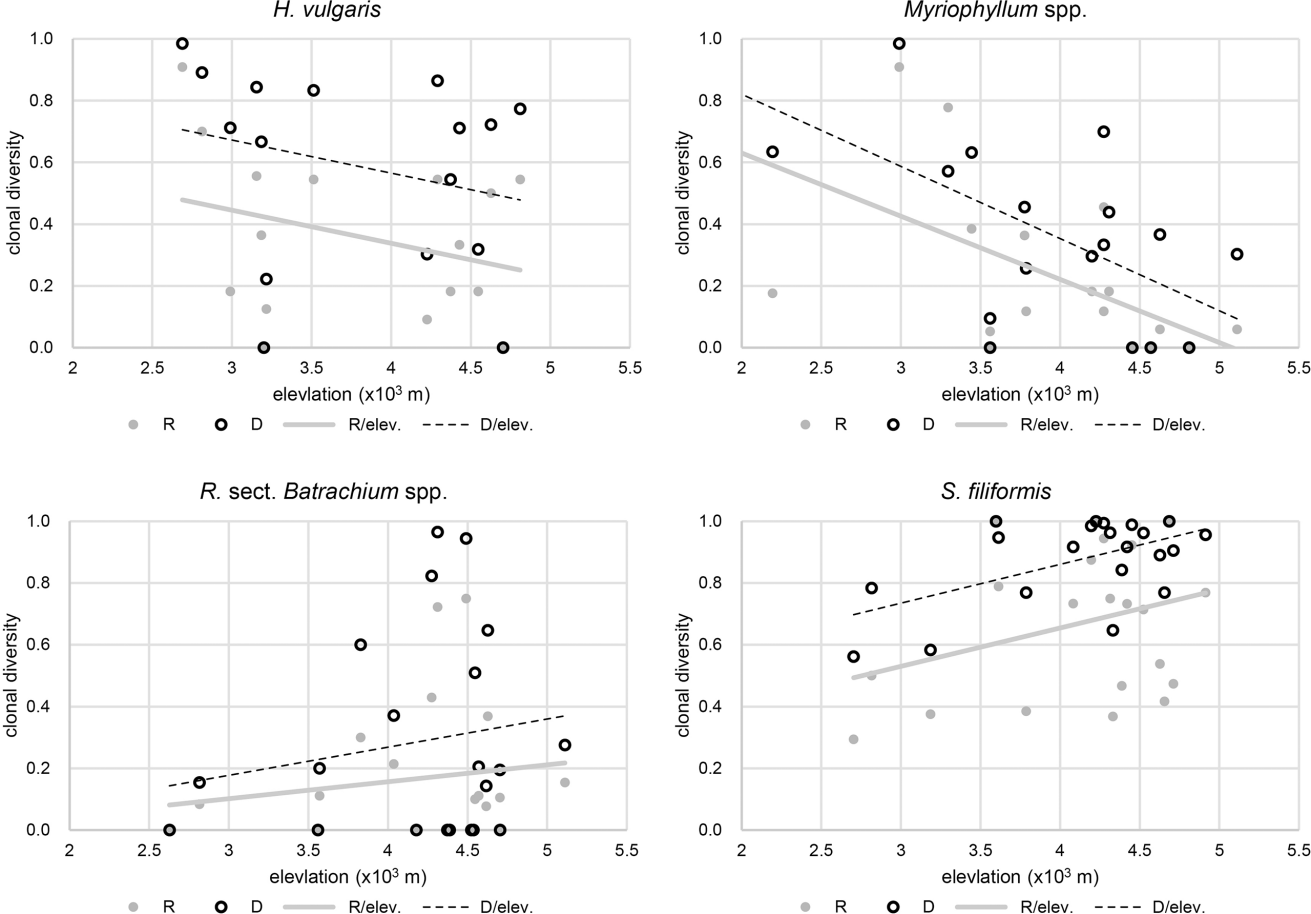
Correlation between clonal diversity and elevational gradient.

The results of beta regression analysis indicated that the environmental effects on clonal diversity were not significant in *R.* sect. *Batrachium* spp. but were significant in the other three taxa (**Table 2**). For *H. vulgaris*, clonal diversity was affected by the local environment, and more than half of clonal variation among the populations was explained by TP and Sal, respectively (**Table 2** and **Figure 3**). TP had a positive effect on the clonal diversity of *H. vulgaris*, while sexual reproduction decreased with increasing Sal (**Table 3**). TN and TS were also suggested by beta regression models as having a significant effect on the clonal variation in *H. vulgaris* (**Table 2**). For *Myriophyllum* spp., elevation and sediment characteristics were important factors influencing the efficiency of sexual recruitment (**Table 2** and **Figure 3**). Elevation constrained sexual reproduction in *Myriophyllum* spp., with the highest relative importance (R: 0.80, D: 0.80) (**Table 3**). Low sedimental nutrition (TN and TP) also had a negative effect on sexual recruitment in *Myriophyllum* spp. (**Table 3**). For *S. filiformis*, climate was the principal factor determining the variations in clonal diversity, and the total variation explained by AT was 16.2% (R) and 22.2% (D)(**Table 2**). A negative relationship between AT and clonal diversity was found in *S. filiformis*, which suggested that the relatively high temperature had a negative impact on sexual reproduction. Elevation and TS were also suggested to be important for clonal variation in *S. filiformis*, while higher D was found in populations with higher elevation and lower temperature seasonality (**Table 3** and **Figure 3**).

**Table 2:** Summary of best models explaining variation in clonal diversity of four aquatic plant taxa on the Qinghai-Tibetan Plateau.

	AIC	ΔAIC	Weight	Pseudo *R* ^2^
**Index R**				
***H. vulgaris***				
TN	0.7		0.453	0.177
TP	1.4	0.7	0.316	0.195
TP+Sal	2	1.3	0.231	0.368
***Myriophyllum* spp.**				
Elev+TN	-11.7		1	0.546
***R. sect. Batrachium* spp.**				
–	–	–	–	–
***S. filiformis***				
AT	-5.6		0.678	0.162
AT+Sal	-4.1	1.5	0.322	0.218
**Index D**				
***H. vulgaris***				
TP+Sal+TS	-0.1		1	0.719
***Myriophyllum* spp.**				
Elev+TN	-3.4		0.412	0.547
Elev+TP+AT	-2.1	1.3	0.221	0.485
Elev+TP	-1.8	1.6	0.188	0.443
Elev	-1.7	1.7	0.179	0.303
***R. sect. Batrachium* spp.**				
–	–	–	–	–
***S. filiformis***				
Elev	-37.8		0.274	0.201
AT+TS	-37.7	0.1	0.255	0.322
AT	-37.6	0.2	0.242	0.222
TS	-37.4	0.4	0.229	0.190

TN, total sediment nitrogen; TP, total sediment phosphorus; Sal, water salinity; AT, annual temperature; TS, temperature seasonality; Elev, elevation. Models were calculated for clonal richness (R) and Simpson complement unbiased clonal diversity (D). The best models with AIC < 2 are presented.

**Table 3:** Relative importance (RI) of environmental factors among all model compilations.

		*H. vulgaris*		*Myriophyllum* spp.		*R. sect. Batrachium* spp.		*S. filiformis*	
		RI (R/D)	DI (R/D)	RI (R/D)	DI (R/D)	RI (R/D)	DI (R/D)	RI (R/D)	DI (R/D)
Elev		0.19/0.11	-/-	**0.80/0.80**	-/-	0.18/0.18	+/+	0.17/**0.33**	+/+
climate	AT	0.15/0.16	+/+	0.19/**0.38**	-/-	0.16/0.15	+/-	**0.45/0.37**	-/-
	TS	0.23/**0.62**	+/+	0.11/0.24	-/+	0.17/0.17	-/-	0.25/**0.46**	-/-
water	pH	0.12/0.06	-/+	0.18/0.15	+/-	0.17/0.18	-/-	0.17/0.15	-/-
	Sal	**0.28/0.79**	-/-	0.09/0.09	+/-	0.20/0.18	-/-	**0.32**/0.18	+/+
sediment	TN	**0.32/**0.10	+/+	**0.61**/**0.40**	+/+	0.15/0.15	-/-	0.16/0.24	-/-
	TP	**0.41**/**0.80**	+/+	0.10/**0.50**	+/+	0.15/0.15	-/-	0.22/0.28	+/+
	TC	0.10/0.07	+/+	0.13/0.08	-/-	0.15/0.17	+/+	0.17/0.28	+/+

Elev, elevation; AT, annual temperature; TS, temperature seasonality; Sal, water salinity; TN, total sediment nitrogen; TP, total sediment phosphorus; TC, total sediment carbon; ‘+’ positive relation; ‘-’ negative relation. Direction of influence (DI) between the clonal diversity (index R and D) and the explanatory variable. Bold values indicate the factors selected in best models.

## Discussion

### Clonal and genetic diversity of aquatic plants in highlands

With the availability of molecular markers with high levels of polymorphism, it has been recognized that clonal plants are not as genetically monomorphic as originally thought (Arnaud-Haond et al., 2005; Vallejo-Marín et al., 2010). Generally, the mean values of clonal diversity (G/N) summarized by multiple studies have been between 0.4 and 0.5 (Arnaud-Haond et al., 2007; Honnay and Jacquemyn, 2008; Vallejo-Marín et al., 2010). Empirical studies have indicated that considerable sexual reproduction within clonal populations in some freshwater species (Yakimowski and Barrett, 2014; Eckert et al., 2016), including submerged macrophytes that are able to fragment any branch to a potential propagule (Sculthorpe, 1967; Triest and Fénart, 2013; Li, 2014). Broad-scale genetic uniformity based on extensive clonality is more common in invasive aquatic species than in other aquatic species (Lambertini et al., 2010). Species involved in the present work or their relatives have been found to have abundant sexual reproduction in the plains (Wang, 2009; Triest and Fénart, 2013; Wu et al., 2016). However, whether sexual reproduction is abundant or constrained in highland regions is not clear in previous studies.

The present work is the first study revealing the clonal diversity pattern of aquatic plants on the QTP based on a multispecies investigation. We found that the high-altitude populations of aquatic taxa showed moderate (*H. vulgaris* and *Myriophyllum* spp.) to high (*S. filiformis*) clonal variation, except *R.* sect. *Batrachium* spp. Under adverse environmental conditions, relatively high clonal diversity may also be induced by migration or long-lived genets (Gabrielsen and Brochmann, 1998; Douhovnikoff et al., 2010). For obviously isolated and small populations of aquatic plants on the QTP, clonal diversity was mainly established by sexual recruitment (Bauert, 1996).

In alpine populations, low genetic diversity might be mainly derived from limited sexual reproduction and selfing or genetic drift induced by severe isolation (Fischer et al., 2000). In the present work, we found a significantly positive correlation between clonal and genetic diversity in relative taxon, except in *H. vulgaris*, indicating that sexual recruitment is essential for genetic diversity in aquatic plants on the QTP. The lack of correlation between the two measures of diversity in *H. vulgaris* might be because some of the populations were composed of a few highly heterozygous genets (McLellan et al., 1997). *S. filiformis*, which is most widely distributed on the QTP (Wang, 2003), presents the highest clonal diversity in the present study. It supports the expansion of aquatic plants by seeds might be fundamental, when interpopulation dispersal *via* vegetative propagules might be rare when habitats are seldom connected by water flows on the QTP (Silvertown, 2008). Although our study did not address adaptive strategies or mechanisms, moderate to high levels of clonal and genetic diversity suggested that these plants cope with the harsh alpine environment not solely by cloning but also by adapting based on sexual breeding (Richards et al., 2004). Our data revealed that sexual reproduction could be important in maintaining aquatic diversity in stressful habitats at local and regional scales.

### Variation in clonal diversity among aquatic taxa

Among the four taxa, *R.* sect. *Batrachium* spp. had significantly lower clonal diversity, and self-compatibility was widely revealed in this species, with efficient outcrossing in a short time (Webster, 1984). The sexual recruitment in *R.* sect. *Batrachium* has been observed in the subalpine regions of the southeast edge of the QTP (Wang, 2009). In the absence of flower visitors, *R.* sect. *Batrachium* may turn towards cleistogamy in response to adverse environments (Hong, 1991). In addition, in alpine areas, autonomous selfing occurs for a “better than nothing” strategy, but inbreeding depression and the accumulation of harmful somatic mutations are more serious (Zhang et al., 2014). We also identified low genetic diversity in *R.* sect. *Batrachium* spp. on the QTP, while the high clonal diversity of a few populations located at elevations between 4000 and 5000 m might be derived from occasional outcrossing, which requires further studies to determine the beneficial factors.

In contrast, dichogamy and wind pollination of *S. filiformis*, *H. vulgaris* and *Myriophyllum* spp. provide the advantage of easy pollen dispersal and low investment in sexual reproduction in the alpine region (Sun et al., 2014). These traits are well adapted to alpine environments to improve their reproductive success on the QTP (Sun et al., 2014), suggesting that the differences in breeding system and life history among the four taxa were the determinants of the clonal diversity of these aquatic plants on the QTP. In *S. latifolia*, the great influence of sexual systems on clonal structure and diversity was also supported, when genotypic richness was significantly higher in dioecious populations (mean R=0.62) than in monoecious populations (mean R = 0.42) (Yakimowski and Barrett, 2014).

Different genetic clades within species may also exhibit obvious differences in clonal diversity (Eckert, 2002). The genetic divergence within species arose from historical movement and environmental heterogeneity (Ohsawa and Ide, 2008). Kimura et al. (2013) found a divergence of clonality in district genetic clusters caused by the snow depth condition for the tree species *Cryptomeria japonica*. Previously, we revealed that the genetic clustering of *R. subrigidus* and *S. filiformis* was affected by environmental conditions (Wu et al., 2019; Wu et al., 2020), but differences in clonal diversity among genetic clusters were not significant in either taxon.

### Influence of altitude on clonal diversity

Sexual reproduction through seeds is threatened in alpine environments by low temperature, short growing season, flooding in summer, strong wind, and lack of pollinators or disseminators, which lead to the failure of mating, seed maturity and seedling establishment (Körner, 2003; Sun et al., 2014). In high elevation regions, which are always the distribution margin of natural populations, limited founders during recolonization and severe habitat fragmentation and isolation could result in the dominance of a few genets in aquatic environments (Dorken and Eckert, 2001; Eckert et al., 2016). Although asexual reproduction and local spread through clonal growth are common in alpine environments (Körner, 2003), sexual reproduction and outcrossing can improve the quality of seeds and the genetic diversity of offspring, which are beneficial and crucial to the maintenance of populations under complex and changing climatic conditions in highlands (Fabbro and Körner, 2004). Thus, the trends of clonal variation with increasing altitude vary in different taxa (Ohsawa and Ide, 2008; Vázquez-Ramírez and Venn, 2021). Low genotypic richness has been suggested in many alpine plants (Reisch et al., 2007; Reisch et al., 2020), while genetic diversity is unaffected or even promoted by higher elevations, which has also been supported by empirical studies, particularly in herbs (Ohsawa and Ide, 2008; Carbognani et al., 2019).

We found that the influence of high altitude on clonal variation was different among the four studied taxa. The effect of elevation on the balance of sexual and asexual reproduction was not significant in *H. vulgaris* and *R.* sect. *Batrachium* spp., while a significant reduction in clonal diversity at higher elevations was only found in *Myriophyllum* spp., especially in areas above 4500 m. Based on field work, we observed entire and expanded bracts (generally subdivided and degenerated) in *M. sibiricum*, implying that the plant might have evolved to invest more in producing flowers against adverse conditions (e.g., strong wind) (Aiken et al., 1979). We also observed a considerable germination rate and early florescence by transplanting *Myriophyllum* plants to central China (Wuhan, 30.26°N, 114.56°E). These findings suggested a decline in clonal diversity along the altitudinal gradient in *Myriophyllum* spp. might be caused by deficiency in seedling establishment.

In *S. filiformis*, clonal diversity was revealed to decline at lower elevational regions, which was mainly caused by annual temperature. *Stuckenia* spp. are abundant in mountain regions of Central Asia and are highly adaptable to alpine environments, and *S. filiformis* occurs almost in cold and shallow water in mountains or in Siberia and is peripheral and unsuitable in low-altitude areas of the plateau edge, which mainly lead to the limitation of its sexual reproduction (Kaplan, 2008). A similar situation was found at the southern edge populations in the arctic-alpine species *Salix herbacea*, and the impact of peripherality on clonal variation was identified (Carbognani et al., 2019). The results are consistent the findings that some arctic-alpine plants increase clonal growth and decrease sexual investment under warmer temperatures (Elmendorf et al., 2012; Frei et al., 2014).

### Influence of the local environment on clonal diversity

Compared to broad-scale environmental heterogeneity, how the local environment affects the proportion of asexual reproduction among populations of clonal plants has received less attention. The trade-off between sexual and asexual reproduction shifts under different local conditions, such as water depth, in aquatic plants (Thompson and Eckert, 2004; Van Drunen and Dorken, 2012; Li et al., 2018). In *Carex* spp., more investment in sexual reproduction than in the asexual reproduction modes was revealed in fertile habitats (Reisch et al., 2020). In fact, the nutrition content of soil or sediment is one of most important factors that changes the balance between clonal and sexual reproduction in plants. The availability of nutrients not only determines the trade-off between the two reproduction modes but also has a strong impact on seed recruitment, especially seedling establishment (Reisch et al., 2020). Previous studies highlighted the greater importance of phosphorus. Extremely low clonal diversity was revealed in *Convallaria majalis*, with only an exceptional population located at the site with abundant soil phosphorus (Vandepitte et al., 2010).

In the present work, we also revealed that local environments have significant effects on the sexual reproduction of aquatic plants on the QTP, in addition to climatic factors. Compared to other regions in China, the QTP has relatively high soil TP contents (Han et al., 2005), and we found that higher TP benefited sexual recruitment in *H. vulgaris* and *Myriophyllum* spp. in the highlands. In nutrient-poor water bodies, the community structure of aquatic macrophytes may also be limited by nitrogen availability. Although plants acquire nitrogen in a variety of ways, the effect of nitrogen availability on the trade-offs between two reproduction modes has been investigated by manipulation experiments (Reekie, 1991). The TN content of the studied sites varied greatly in the present work, and nitrogen limitation of aquatic macrophytes on the QTP has been suggested (Wang et al., 2015), and based on our results, it was also suggested that the clonal variation of *H. vulgaris* and *Myriophyllum* spp. is constrained by TN.

The effects of water characteristics on the reproduction of aquatic plants have received less attention. pH determines the form of inorganic carbon in water bodies, subsequently affecting the photosynthesis and productivity of submerged macrophytes (Barko et al., 1986). The availability of free CO_2_ and HCO_3_^–^ is constrained with increasing pH (Bowes, 1987). In the present work, we did not find evidence for a negative correlation between clonal diversity and pH in either taxon. However, at pH>10, when inorganic carbon (mainly as CO_3_^2-^) was rarely used directly by submerged plants, most populations were monomorphic in *R.* sect. *Batrachium* and *Myriophyllum* spp., implying that water pH might affect the reproduction of some aquatic plants above a certain threshold.

*Myriophyllum* spp. and *S. filiformis* were sampled at the five sites with salinities over 1%, and the clonal diversity of these populations was equal to the mean value of related taxon. Although seedling establishment of plants could be limited in habitats with high salinity (Adam, 1990), adaptative life history strategies to saline environments might result in high clonal diversity in dominant salt marsh plants (Richards et al., 2004; Tumas et al., 2019). For freshwater plants that tolerate a certain level of salinity, few studies have focused on their reproductive patterns related to water salinity. The results of this study did not support that high salinity constrains sexual reproduction of the two taxa. (Li et al., 2015) also found that the seed weight and germination rates of *S. pectinata*, which is closely related to *S. filiformis*, is unaffected by water salinity but not pH. Because few populations have been sampled in water with a high pH or salinity level, the influences of water chemistry on clonal reproduction need further study.

## Conclusions

According to our results, we revealed different patterns of clonal diversity in four aquatic taxa along an elevational gradient on the QTP. In general, clonal diversity was moderate to high in aquatic plants in the highlands except in *R.* sect. *Batrachium* spp., indicating the importance of sexual recruitment on the maintenance of aquatic macrophytes in the highlands. The effects of elevational gradients on the genetic diversity of the aquatic taxa were different. High elevation only constrained sexual reproduction in *Myriophyllum* spp. At lower altitudes, *S. filiformis* tended to undergo vegetative growth and reproduction, possibly because of peripheral effects on arc-alpine species in warmer regions. The clonal diversity was also positively correlated with nutrition availability in *H. vulgaris* and *Myriophyllum* spp., suggesting the obvious influence of local habitat on the genetic pattern in aquatic plants. We found little evidence of the effects of water characteristics on the sexual reproduction of aquatic plants, which requires further studies based on more sample sites with a high pH and salinity level. In the present work, we highlighted the great influence of sexual systems and environments on clonal structure and genetic diversity on the QTP.

## Data availability statement

The original contributions presented in the study are included in the article/**Supplementary Materials**. Further inquiries can be directed to the corresponding authors.

## Author contributions

ZWU, XX, TL and JZ designed the study. ZWU, ZWA, AZ, YW and HAW performed the field work and experiments. ZWU, ZWA, DX and HUW analyzed the data. ZWU and XX wrote the manuscript. All authors contributed to the article and approved the submitted version.

## Funding

This research was supported by the Strategic Priority Research Program of Chinese Academy of Sciences (XDB31000000), the National Natural Science Foundation of China (31700190) and Special Foundation of President of The Chinese Academy of Sciences.

## Acknowledgments

The authors thank Haocun Zhao for the assistance on field work, and Qixiang Lu, Xing Li, Juan Zhang for the valuable suggestion. We are also grateful to reviewers for their very helpful comments and advices.

## Conflict of interest

The authors declare that the research was conducted in the absence of any commercial or financial relationships that could be construed as a potential conflict of interest.

## Publisher’s note

All claims expressed in this article are solely those of the authors and do not necessarily represent those of their affiliated organizations, or those of the publisher, the editors and the reviewers. Any product that may be evaluated in this article, or claim that may be made by its manufacturer, is not guaranteed or endorsed by the publisher.
