# Supplementary material for: Effects of highland environments on clonal diversity in aquatic plants: An interspecific comparison study on the Qinghai-Tibetan Plateau
Source: Front Plant Sci. 2022 Oct 20;13:1040282. doi: 10.3389/fpls.2022.1040282 (PMC9632175; doi:10.3389/fpls.2022.1040282)
Supplement: Supplementary file 2 [file DataSheet_2.docx]

**Supplementary Material**


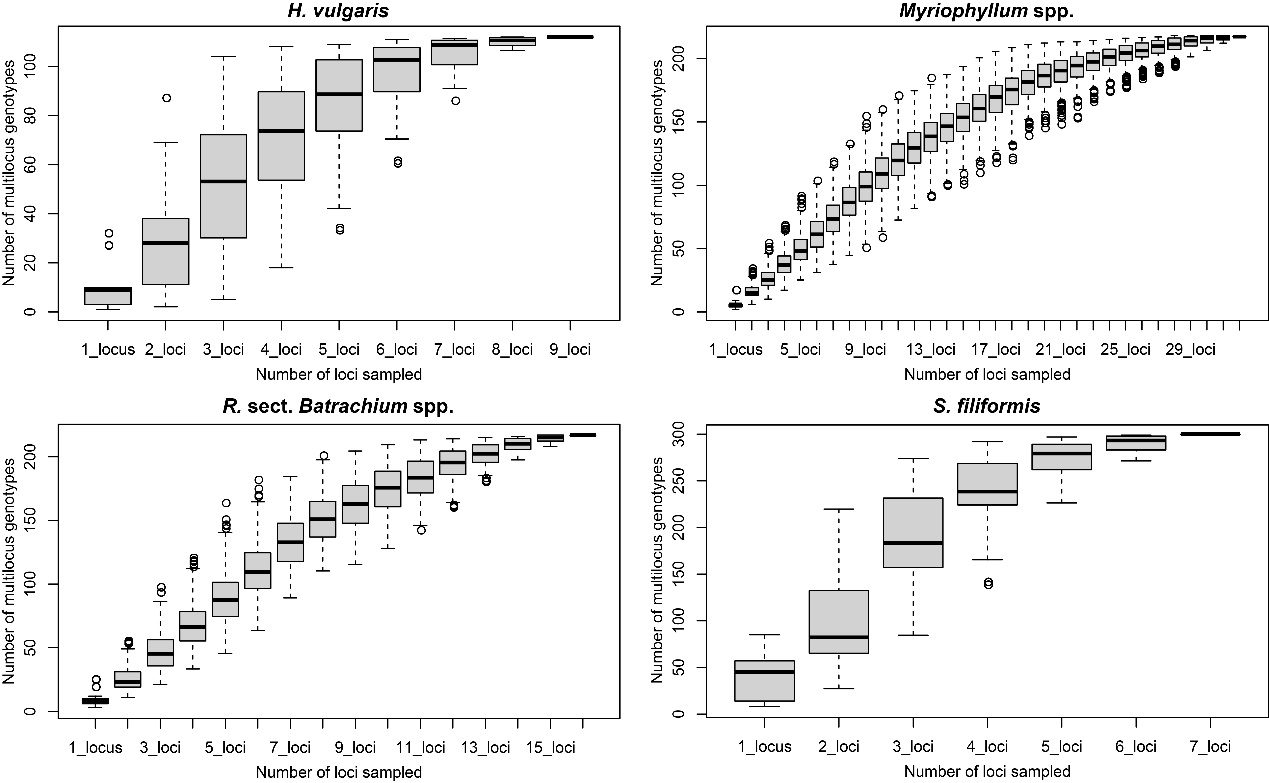


**Figure 1** Genotype accumulation curve for reliability of subsampling of ramets and number of loci. The multilocus data of the polyploid *Myriophyllum* spp. was converted into binary form based on the presence/absence of observed alleles.


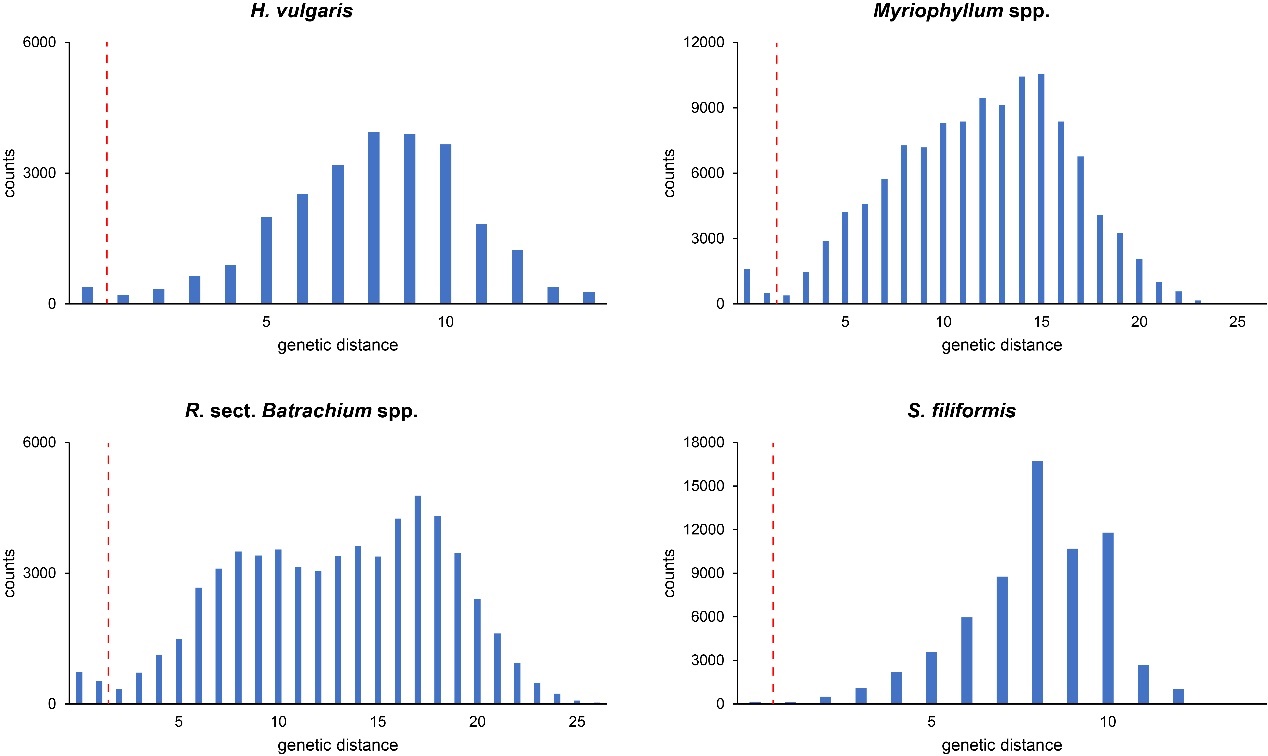


**Figure 2** Frequency distribution of pairwise genetic distance between observed ramets in relative taxon, the threshold is indicated by the vertical red line.


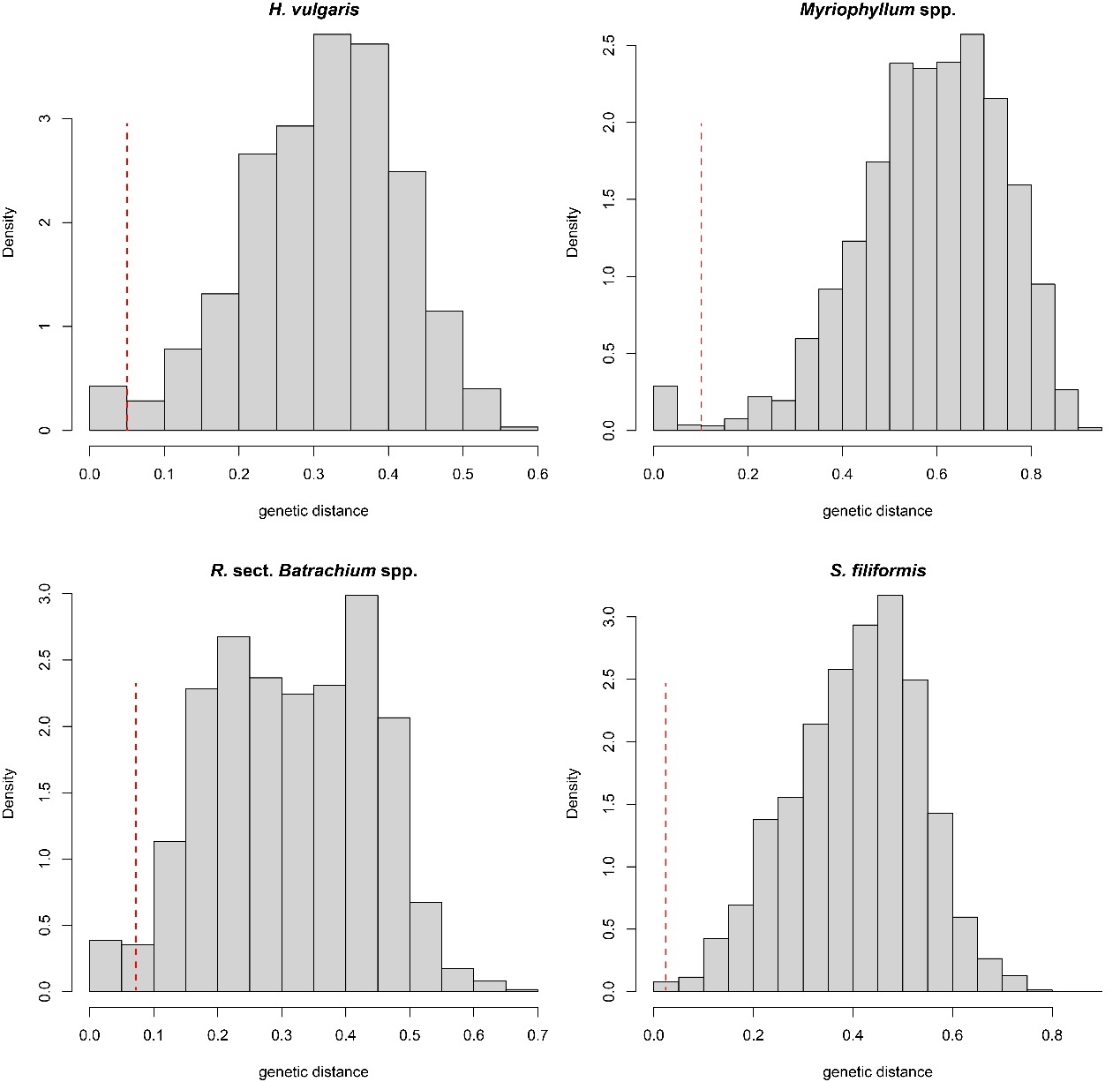


**Figure 3** Frequency distribution of pairwise Bruvo distance between all ramets, and threshold distance for relative taxon is depicted.

**
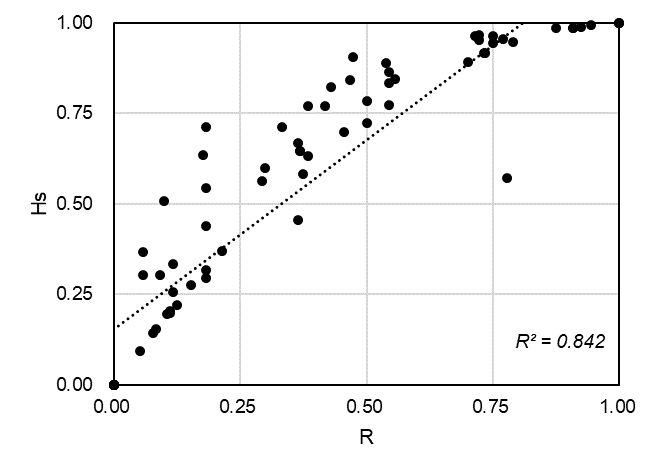
**

**Figure 4** Correlation between clonal diversity index R and D





**Figure 5** Correlation between clonal diversity and genetic diversity for relative taxon


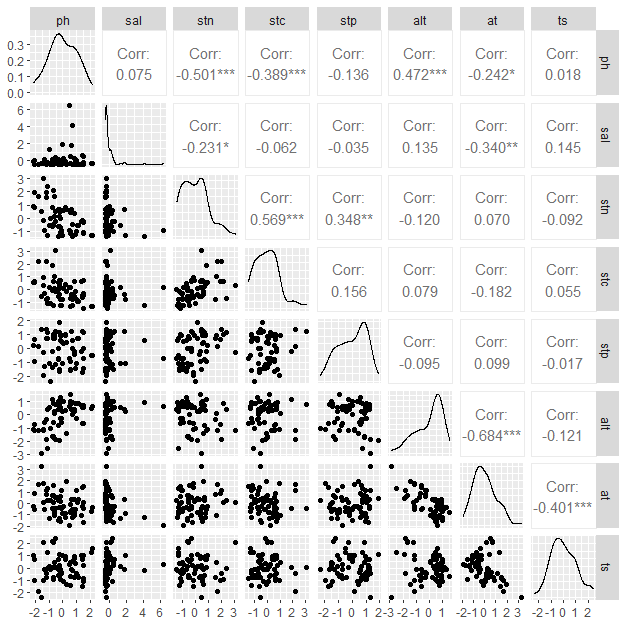


**Figure 6** Pearson’s correlation among environmental variables

**Table 1.** Geographic information and genetic diversity of all studied populations.

| Taxon | Lineage | Lat | Long | Elev | nr | ng | ng.b | R | D | Hs |
| --- | --- | --- | --- | --- | --- | --- | --- | --- | --- | --- |
| *H. vulgaris* | LineageB2 | 27.5 | 99.82 | 3.185 | 12 | 5 | 5 | 0.364 | 0.667 | 0.131 |
| *H. vulgaris* | LineageB3 | 28.06 | 91.95 | 4.627 | 9 | 5 | 7 | 0.500 | 0.722 | 0.22 |
| *H. vulgaris* | LineageB2 | 28.59 | 86.83 | 4.373 | 12 | 3 | 4 | 0.182 | 0.545 | 0.365 |
| *H. vulgaris* | LineageB2 | 29.42 | 85.24 | 4.703 | 16 | 1 | 1 | 0.000 | 0.000 | 0.33 |
| *H. vulgaris* | LineageB2 | 29.63 | 94.38 | 2.99 | 12 | 3 | 4 | 0.182 | 0.712 | 0.226 |
| *H. vulgaris* | LineageB3 | 30.02 | 97.04 | 3.201 | 12 | 1 | 1 | 0.000 | 0.000 | 0.056 |
| *H. vulgaris* | LineageB2 | 30.16 | 101.5 | 3.513 | 12 | 7 | 6 | 0.545 | 0.833 | 0.275 |
| *H. vulgaris* | LineageB2 | 30.48 | 91.1 | 4.291 | 12 | 7 | 7 | 0.545 | 0.864 | 0.308 |
| *H. vulgaris* | LineageB3 | 30.75 | 88.79 | 4.81 | 12 | 7 | 7 | 0.545 | 0.773 | 0.187 |
| *H. vulgaris* | LineageB3 | 31.73 | 94.49 | 4.547 | 12 | 3 | 3 | 0.182 | 0.318 | 0.23 |
| *H. vulgaris* | LineageB2 | 33.17 | 79.84 | 4.429 | 10 | 4 | 4 | 0.333 | 0.711 | 0.322 |
| *H. vulgaris* | LineageB3 | 33.26 | 103.76 | 2.689 | 12 | 11 | 11 | 0.909 | 0.985 | 0.114 |
| *H. vulgaris* | LineageB3 | 34.72 | 98.1 | 4.225 | 12 | 2 | 3 | 0.091 | 0.303 | 0.337 |
| *H. vulgaris* | LineageB2 | 37.25 | 97.03 | 2.812 | 11 | 8 | 8 | 0.700 | 0.891 | 0.294 |
| *H. vulgaris* | LineageB2 | 37.54 | 95.43 | 3.155 | 10 | 6 | 6 | 0.556 | 0.844 | 0.152 |
| *H. vulgaris* | LineageB3 | 37.61 | 101.32 | 3.216 | 9 | 2 | 2 | 0.125 | 0.222 | 0.142 |
| *Myriophyllum* spp. | *M. spicatum* | 25.67 | 100.21 | 1.954 | 19 | 14 | 14 | 0.722 | 0.953 | 0.182 |
| *Myriophyllum* spp. | *M. sibiricum* | 28.49 | 98.91 | 3.56 | 13 | 1 | 1 | 0.000 | 0.000 | 0.024 |
| *Myriophyllum* spp. | *M. spicatum* | 28.77 | 91.67 | 4.626 | 18 | 2 | 2 | 0.059 | 0.366 | 0.049 |
| *Myriophyllum* spp. | *M. spicatum* | 29.04 | 100.3 | 3.776 | 12 | 5 | 5 | 0.364 | 0.455 | 0.23 |
| *Myriophyllum* spp. | *M. spicatum* | 29.21 | 87.42 | 4.307 | 12 | 3 | 5 | 0.182 | 0.439 | 0.089 |
| *Myriophyllum* spp. | *M. spicatum* | 29.32 | 89.41 | 3.787 | 18 | 3 | 5 | 0.118 | 0.257 | 0.09 |
| *Myriophyllum* spp. | *M. spicatum* | 29.63 | 94.38 | 2.99 | 12 | 11 | 11 | 0.909 | 0.985 | 0.101 |
| *Myriophyllum* spp. | *M. sibiricum* | 29.64 | 96.72 | 4.453 | 17 | 1 | 1 | 0.000 | 0.000 | 0.051 |
| *Myriophyllum* spp. | *M. sibiricum* | 29.69 | 85.72 | 5.111 | 18 | 2 | 2 | 0.059 | 0.303 | 0.286 |
| *Myriophyllum* spp. | *M. spicatum* | 29.7 | 84.13 | 4.569 | 14 | 1 | 1 | 0.000 | 0.000 | 0.008 |
| *Myriophyllum* spp. | *M. spicatum* | 29.89 | 93.46 | 3.56 | 20 | 2 | 1 | 0.053 | 0.095 | 0.033 |
| *Myriophyllum* spp. | *M. spicatum* | 30.75 | 88.79 | 4.81 | 18 | 1 | 1 | 0.000 | 0.000 | 0.023 |
| *Myriophyllum* spp. | *M. sibiricum* | 32.44 | 102.37 | 3.444 | 14 | 6 | 6 | 0.385 | 0.632 | 0.141 |
| *Myriophyllum* spp. | *M. sibiricum* | 33.56 | 102.48 | 3.297 | 10 | 8 | 8 | 0.778 | 0.571 | 0.241 |
| *Myriophyllum* spp. | *M. sibiricum* | 34.85 | 98.13 | 4.2 | 12 | 3 | 2 | 0.182 | 0.296 | 0.114 |
| *Myriophyllum* spp. | *M. spicatum* | 34.86 | 97.49 | 4.274 | 18 | 3 | 3 | 0.118 | 0.333 | 0.116 |
| *Myriophyllum* spp. | *M. sibiricum* | 34.86 | 97.49 | 4.274 | 12 | 6 | 6 | 0.455 | 0.699 | 0.161 |
| *Myriophyllum* spp. | *M. spicatum* | 36.11 | 101.52 | 2.194 | 18 | 4 | 4 | 0.176 | 0.634 | 0.076 |
| *R.* sect. *Batrachium* spp. | *R. trichophyllus* | 28.33 | 87.78 | 4.178 | 11 | 1 | 1 | 0.000 | 0.000 | 0.323 |
| *R.* sect. *Batrachium* spp. | *R. subrigidus* | 28.59 | 86.83 | 4.373 | 20 | 1 | 3 | 0.000 | 0.000 | 0.094 |
| *R.* sect. *Batrachium* spp.*.* | *R. subrigidus* | 28.77 | 91.67 | 4.626 | 20 | 8 | 8 | 0.368 | 0.647 | 0.234 |
| *R.* sect. *Batrachium* spp. | *R. trichophyllus* | 28.78 | 92.09 | 4.49 | 9 | 7 | 5 | 0.750 | 0.944 | 0.324 |
| *R.* sect. *Batrachium* spp. | *R. trichophyllus* | 29.25 | 91.4 | 3.57 | 11 | 2 | 2 | 0.111 | 0.200 | 0.218 |
| *R.* sect. *Batrachium* spp. | *R. subrigidus* | 29.42 | 85.24 | 4.703 | 20 | 3 | 3 | 0.105 | 0.195 | 0.079 |
| *R.* sect. *Batrachium* spp. | *R. trichophyllus* | 29.69 | 85.72 | 5.111 | 14 | 3 | 4 | 0.154 | 0.275 | 0.325 |
| *R.* sect. *Batrachium* spp. | *R. subrigidus* | 29.7 | 84.13 | 4.569 | 19 | 3 | 5 | 0.111 | 0.205 | 0.158 |
| *R.* sect. *Batrachium* spp. | *R. trichophyllus* | 29.84 | 91.72 | 3.828 | 16 | 4 | 4 | 0.300 | 0.600 | 0.338 |
| *R.* sect. *Batrachium* spp. | *R. trichophyllus* | 29.89 | 93.46 | 3.56 | 21 | 1 | 3 | 0.000 | 0.000 | 0.213 |
| *R.* sect. *Batrachium* spp. | *R. subrigidus* | 30 | 89.1 | 4.311 | 19 | 14 | 12 | 0.722 | 0.965 | 0.243 |
| *R.* sect. *Batrachium* spp. | *R. trichophyllus* | 31.68 | 94.88 | 4.536 | 14 | 1 | 2 | 0.000 | 0.000 | 0.315 |
| *R.* sect. *Batrachium* spp. | *R. trichophyllus* | 31.73 | 94.49 | 4.547 | 10 | 2 | 2 | 0.100 | 0.509 | 0.198 |
| *R.* sect. *Batrachium* spp. | *R. subrigidus* | 31.89 | 80.16 | 4.387 | 21 | 1 | 1 | 0.000 | 0.000 | 0.097 |
| *R.* sect. *Batrachium* spp. | *R. subrigidus* | 32.1 | 81.79 | 4.616 | 14 | 2 | 2 | 0.077 | 0.143 | 0.133 |
| *R.* sect. *Batrachium* spp. | *R. subrigidus* | 32.4 | 80.83 | 4.524 | 14 | 1 | 1 | 0.000 | 0.000 | 0.154 |
| *R.* sect. *Batrachium* spp. | *R. subrigidus* | 33.91 | 99.56 | 4.036 | 15 | 4 | 6 | 0.214 | 0.371 | 0.134 |
| *R.* sect. *Batrachium* spp. | *R. subrigidus* | 34.86 | 97.49 | 4.274 | 22 | 10 | 10 | 0.429 | 0.823 | 0.202 |
| *R.* sect. *Batrachium* spp. | *R. subrigidus* | 35.15 | 93.04 | 4.704 | 16 | 1 | 2 | 0.000 | 0.000 | 0.098 |
| *R.* sect. *Batrachium* spp. | *R. subrigidus* | 37.1 | 101.57 | 2.628 | 15 | 1 | 2 | 0.000 | 0.000 | 0.098 |
| *R.* sect. *Batrachium* spp. | *R. subrigidus* | 37.25 | 97.03 | 2.816 | 13 | 2 | 2 | 0.083 | 0.154 | 0.094 |
| *S. filiformis* | Clade2 | 28.36 | 86.5 | 4.314 | 17 | 13 | 14 | 0.750 | 0.963 | 0.403 |
| *S. filiformis* | Clade2 | 28.46 | 91.4 | 4.626 | 14 | 11 | 13 | 0.538 | 0.890 | 0.412 |
| *S. filiformis* | Clade2 | 29.19 | 89.25 | 3.787 | 14 | 8 | 10 | 0.385 | 0.769 | 0.232 |
| *S. filiformis* | Clade2 | 29.44 | 91.25 | 3.613 | 20 | 16 | 19 | 0.789 | 0.947 | 0.365 |
| *S. filiformis* | Clade3 | 31.14 | 85.05 | 4.685 | 13 | 13 | 13 | 1.000 | 1.000 | 0.477 |
| *S. filiformis* | Clade2 | 31.15 | 88.06 | 4.655 | 13 | 6 | 8 | 0.417 | 0.769 | 0.523 |
| *S. filiformis* | Clade2 | 31.26 | 80.17 | 4.913 | 14 | 6 | 8 | 0.769 | 0.956 | 0.232 |
| *S. filiformis* | Clade3 | 31.53 | 80.1 | 4.387 | 16 | 8 | 9 | 0.467 | 0.842 | 0.396 |
| *S. filiformis* | Clade2 | 32.08 | 84.43 | 4.421 | 16 | 12 | 12 | 0.733 | 0.917 | 0.339 |
| *S. filiformis* | Clade2 | 32.17 | 89.05 | 4.711 | 20 | 17 | 15 | 0.474 | 0.905 | 0.471 |
| *S. filiformis* | Clade1 | 32.17 | 96.28 | 3.598 | 17 | 10 | 10 | 1.000 | 1.000 | 0.558 |
| *S. filiformis* | Clade2 | 32.24 | 80.49 | 4.524 | 15 | 11 | 11 | 0.714 | 0.962 | 0.4 |
| *S. filiformis* | Clade3 | 32.26 | 83.1 | 4.451 | 14 | 13 | 13 | 0.923 | 0.989 | 0.529 |
| *S. filiformis* | Clade3 | 33.1 | 79.51 | 4.331 | 20 | 8 | 10 | 0.368 | 0.647 | 0.323 |
| *S. filiformis* | Clade1 | 33.2 | 97.25 | 4.195 | 17 | 15 | 16 | 0.875 | 0.985 | 0.438 |
| *S. filiformis* | Clade1 | 34.09 | 100.09 | 4.082 | 16 | 12 | 15 | 0.733 | 0.917 | 0.333 |
| *S. filiformis* | Clade3 | 34.41 | 98.07 | 4.225 | 17 | 17 | 17 | 1.000 | 1.000 | 0.517 |
| *S. filiformis* | Clade1 | 34.52 | 97.3 | 4.274 | 19 | 18 | 19 | 0.944 | 0.994 | 0.563 |
| *S. filiformis* | Clade1 | 37.15 | 97.02 | 2.816 | 19 | 14 | 17 | 0.500 | 0.784 | 0.263 |
| *S. filiformis* | Clade1 | 37.37 | 101.19 | 3.186 | 9 | 4 | 5 | 0.375 | 0.583 | 0.263 |
| *S. filiformis* | Clade1 | 38.13 | 94.22 | 2.703 | 18 | 6 | 6 | 0.294 | 0.562 | 0.353 |

Lat, latitude; Long, longitude; Elev, elevation range (x10^3^ m); nr: number of ramets; ng, number of genets; ng.b, number of genets estimated using poppr/polysat based on Bruvo distance; R, index of clonal richness; D, Simpson complement unbiased clonal diversity; Hs, heterozygosity within populations

**Table 2.** Differences in clonal diversity among genetic lineages for each taxon.

| Taxon | Lineage | *p*-value | |
| --- | --- | --- | --- |
|  |  | R | D |
| *H. vulgaris* | Lineage B2/B3 | 0.488 | 0.368 |
| *Myriophyllum* spp. | *M. spicatum*/*M.sibiricum* | 0.855 | 0.855 |
| *R.* sect. *Batrachium* spp. | *R. subrigidus*/*R. trichophyllus* | 0.852 | 0.765 |
| *S. filiformis* | Clade1/2/3 | 0.656 | 0.604 |

**Table 3.** Local environmental characteristics for each sample site.

| Lat | Long | Elev | ph | Sal | TN | TC | TP | AT | TS |
| --- | --- | --- | --- | --- | --- | --- | --- | --- | --- |
| 25.67 | 100.21 | 1.954 | 7.81 | 0.16 | 3.89 | 30.24 | 0.77 | 157 | 4242 |
| 27.5 | 99.82 | 3.185 | 7.64 | 0.08 | 5.99 | 94.53 | 0.55 | 85 | 4825 |
| 28.06 | 91.95 | 4.627 | 8.48 | 0.1 | 6.83 | 58.83 | 0.71 | 15 | 6006 |
| 28.33 | 87.78 | 4.178 | 9.66 | 0.05 | 3.61 | 30.83 | 0.45 | 27 | 6178 |
| 28.36 | 86.5 | 4.314 | 8.86 | 0.1 | 3.92 | 60.51 | 0.7 | -52 | 5952 |
| 28.46 | 91.4 | 4.626 | 9.84 | 0.04 | 2.05 | 21.11 | 0.66 | 33 | 6181 |
| 28.49 | 98.91 | 3.56 | 10.07 | 0.1 | 2.75 | 28.98 | 0.21 | 59 | 5299 |
| 28.59 | 86.83 | 4.373 | 8.86 | 0.1 | 2.92 | 60.51 | 0.7 | 22 | 6522 |
| 28.77 | 91.67 | 4.626 | 9.78 | 0.04 | 2.05 | 21.11 | 0.66 | 17 | 6335 |
| 28.78 | 92.09 | 4.49 | 8.82 | 0.1 | 4.23 | 48.98 | 0.73 | 24 | 6332 |
| 29.04 | 100.3 | 3.776 | 8.12 | 0.08 | 5.79 | 24.89 | 0.65 | 47 | 6133 |
| 29.19 | 89.25 | 3.787 | 8.87 | 0.14 | 1.02 | 13.92 | 0.71 | 34 | 6700 |
| 29.21 | 87.42 | 4.307 | 9.48 | 1.49 | 4.17 | 34.91 | 0.75 | 23 | 6927 |
| 29.25 | 91.4 | 3.57 | 8.49 | 0.11 | 2.15 | 14.89 | 0.65 | 81 | 6232 |
| 29.32 | 89.41 | 3.787 | 8.91 | 0.15 | 1.12 | 13.83 | 0.7 | 64 | 6581 |
| 29.42 | 85.24 | 4.703 | 9.03 | 0.55 | 4.01 | 45.76 | 0.59 | 0 | 5777 |
| 29.44 | 91.25 | 3.613 | 8.93 | 0.07 | 1.34 | 14.19 | 0.62 | 3 | 6646 |
| 29.63 | 94.38 | 2.99 | 9.17 | 0.05 | 4.19 | 40.54 | 0.61 | 96 | 5462 |
| 29.64 | 96.72 | 4.453 | 10.14 | 0.05 | 1.07 | 35.68 | 0.27 | 13 | 5754 |
| 29.69 | 85.72 | 5.111 | 9.39 | 0.11 | 3.76 | 46.98 | 0.36 | -23 | 6293 |
| 29.7 | 84.13 | 4.569 | 9.43 | 0.17 | 0.89 | 13.28 | 0.24 | 8 | 5381 |
| 29.84 | 91.72 | 3.828 | 9.77 | 0.35 | 1.22 | 25.6 | 0.33 | 58 | 6581 |
| 29.89 | 93.46 | 3.56 | 10.15 | 0.23 | 0.92 | 7.87 | 0.4 | 70 | 6172 |
| 30 | 89.1 | 4.311 | 8.75 | 0.11 | 0.49 | 42.9 | 0.4 | 22 | 6988 |
| 30.02 | 97.04 | 3.201 | 7.94 | 0.12 | 2.75 | 65.45 | 0.17 | 78 | 5872 |
| 30.16 | 101.5 | 3.513 | 7.44 | 0.06 | 6.46 | 58.25 | 0.64 | 50 | 5860 |
| 30.48 | 91.1 | 4.291 | 8.17 | 0.12 | 7.28 | 26.85 | 0.74 | 26 | 7072 |
| 30.75 | 88.79 | 4.81 | 8.42 | 0.2 | 4.53 | 87.27 | 0.48 | -2 | 7172 |
| 31.14 | 85.05 | 4.685 | 9.52 | 3.03 | 0.6 | 14.1 | 0.63 | -33 | 6790 |
| 31.15 | 88.06 | 4.655 | 8.81 | 0.12 | 3.88 | 60.77 | 0.25 | -56 | 7347 |
| 31.26 | 80.17 | 4.913 | 8.86 | 0.22 | 1.82 | 24.48 | 0.63 | -1 | 6964 |
| 31.53 | 80.1 | 4.387 | 10.11 | 0.7 | 2.57 | 32.43 | 0.74 | -59 | 7391 |
| 31.68 | 94.88 | 4.536 | 8.81 | 0.08 | 1.85 | 55.4 | 0.23 | -6 | 6966 |
| 31.73 | 94.49 | 4.547 | 8.6 | 0.44 | 3.88 | 116.05 | 0.75 | -10 | 7103 |
| 31.89 | 80.16 | 4.387 | 10.1 | 0.72 | 2.53 | 32.57 | 0.75 | -7 | 7923 |
| 32.08 | 84.43 | 4.421 | 8.27 | 1.23 | 1.97 | 47.18 | 0.33 | -40 | 7280 |
| 32.1 | 81.79 | 4.616 | 9.67 | 0.22 | 0.8 | 39.39 | 0.26 | -11 | 7576 |
| 32.17 | 89.05 | 4.711 | 9.44 | 0.64 | 1.26 | 27.39 | 0.25 | -18 | 7682 |
| 32.17 | 96.28 | 3.598 | 7.99 | 0.41 | 1.32 | 36.67 | 0.29 | -14 | 6905 |
| 32.24 | 80.49 | 4.524 | 10.02 | 0.1 | 2.53 | 59.24 | 0.49 | -40 | 8271 |
| 32.26 | 83.1 | 4.451 | 9.38 | 4.52 | 1.37 | 48.02 | 0.4 | -62 | 7481 |
| 32.4 | 80.83 | 4.524 | 10.12 | 0.11 | 2.49 | 59.28 | 0.53 | -8 | 8385 |
| 32.44 | 102.37 | 3.444 | 8.75 | 0.06 | 4.22 | 56.12 | 0.38 | 37 | 6337 |
| 33.1 | 79.51 | 4.331 | 10.57 | 0.14 | 0.67 | 11.63 | 0.44 | -78 | 8729 |
| 33.17 | 79.84 | 4.429 | 10.61 | 0.15 | 0.64 | 11.91 | 0.43 | -8 | 9040 |
| 33.2 | 97.25 | 4.195 | 8.55 | 0.2 | 3.24 | 45.09 | 0.65 | -23 | 7228 |
| 33.26 | 103.76 | 2.689 | 8.08 | 0.1 | 6.54 | 95.58 | 0.77 | 58 | 6535 |
| 33.56 | 102.48 | 3.297 | 8.3 | 0.04 | 3 | 18.3 | 0.1 | 23 | 6972 |
| 33.91 | 99.56 | 4.036 | 9.51 | 0.08 | 2.23 | 25.86 | 0.53 | -12 | 7767 |
| 34.09 | 100.09 | 4.082 | 8.52 | 0.12 | 5.94 | 67.43 | 0.69 | -30 | 7687 |
| 34.41 | 98.07 | 4.225 | 9.14 | 0.28 | 4.34 | 53.02 | 0.71 | -40 | 7960 |
| 34.52 | 97.3 | 4.274 | 9.65 | 0.49 | 3.71 | 59.18 | 0.49 | -33 | 8035 |
| 34.72 | 98.1 | 4.225 | 9.14 | 0.31 | 4.45 | 53.14 | 0.7 | -26 | 8229 |
| 34.85 | 98.13 | 4.2 | 8.97 | 1.56 | 0.71 | 25.83 | 0.33 | -27 | 8317 |
| 34.86 | 97.49 | 4.274 | 9.65 | 0.09 | 3.71 | 59.18 | 0.49 | -27 | 8244 |
| 35.15 | 93.04 | 4.704 | 8.76 | 0.45 | 1.91 | 30.42 | 0.53 | -53 | 7969 |
| 36.11 | 101.52 | 2.194 | 8.13 | 0.25 | 0.65 | 17.3 | 0.66 | 71 | 8633 |
| 37.1 | 101.57 | 2.628 | 8.81 | 0.12 | 3.26 | 34.34 | 0.87 | 32 | 8412 |
| 37.15 | 97.02 | 2.816 | 8.41 | 0.48 | 1.93 | 42.5 | 0.28 | 46 | 9633 |
| 37.25 | 97.03 | 2.816 | 8.47 | 0.47 | 2.04 | 42.62 | 0.68 | 45 | 9671 |
| 37.37 | 101.19 | 3.186 | 7.42 | 0.38 | 4.53 | 56.45 | 0.36 | -37 | 7902 |
| 37.54 | 95.43 | 3.155 | 7.95 | 0.15 | 8.47 | 50.08 | 0.47 | 19 | 9610 |
| 37.61 | 101.32 | 3.216 | 7.4 | 0.33 | 4.63 | 56.35 | 0.56 | 1 | 8355 |
| 38.13 | 94.22 | 2.703 | 8.61 | 0.57 | 2.53 | 40.2 | 0.31 | 35 | 10035 |

Lat, latitude; Long, longitude; Elev, elevation; Sal, water salinity; TN, total sediment nitrogen; TP, total sediment phosphorus; TC, total sediment carbon; AT, annual temperature (BIO1); TS, temperature seasonality (BIO4)
